# Comparative Analysis of 60Co and 192Ir Sources in High Dose Rate Brachytherapy for Cervical Cancer

**DOI:** 10.3390/cancers14194749

**Published:** 2022-09-29

**Authors:** Aiping Wen, Xianliang Wang, Bingjie Wang, Chuanjun Yan, Jingyue Luo, Pei Wang, Jie Li

**Affiliations:** 1 School of Medicine, University of Electronic Science and Technology of China, Chengdu 610054, China; 2 Department of Radiation Oncology, Sichuan Cancer Hospital & Institute, Sichuan Cancer Center, Radiation Oncology Key Laboratory of Sichuan Province, Chengdu 610041, China; 3 Department of Health Technology and Informatics, The Hong Kong Polytechnic University, Hongkong 999077, China; 4 Department of Oncology, The Affiliated Hospital of Southwest Medical University, Luzhou 646000, China

**Keywords:** 60Co, 192Ir, cervical cancer, high-dose-rate brachytherapy

## Abstract

**Simple Summary:**

High-dose-rate (HDR) brachytherapy (BT) is an effective treatment for bulky, middle, and advanced cervical cancer. Both 192Ir and 60Co are radiation sources recommended by the ICRU. Previously, due to the large geometric size of 60Co, 192Ir occupied most of the market share. After continuous technical improvements, the geometry of miniaturized 60Co sources has become comparable to that of 192Ir sources. Another important point is that people suspect that the use of 60Co sources will result in reduced efficacy, increased toxic reactions, and a series of issues. In this paper, we compared 60Co and 192Ir sources for HDR BT in terms of both dosimetry and clinical treatment. The results of reports published on the use of HDR BT for cervical cancer over the past few years as well as our own research show that this treatment is safe and 60Co can be promoted as a good alternative to 192Ir.

**Abstract:**

High-dose-rate (HDR) brachytherapy (BT) is an essential treatment for cervical cancer, one of the most prevalent gynecological malignant tumors. In HDR BT, high radiation doses can be delivered to the tumor target with the minimum radiation doses to organs at risk. Despite the wide use of the small HDR 192Ir source, as the technique has improved, the HDR 60Co source, which has the same miniaturized geometry, has also been produced and put into clinical practice. Compared with 192Ir (74 days), 60Co has a longer half-life (5.3 years), which gives it a great economic advantage for developing nations. The aim of the study was to compare 60Co and 192Ir sources for HDR BT in terms of both dosimetry and clinical treatment. The results of reports published on the use of HDR BT for cervical cancer over the past few years as well as our own research show that this treatment is safe and it is feasible to use 60Co as an alternative source.

## 1. Introduction

High-dose-rate (HDR) brachytherapy (BT) is a significant component of radiotherapy. The unique aspect of this treatment is that the radiation source needs to be placed inside the part to be treated. This ensures a higher radiation dose is transmitted to the center of the tumor target, while the dose given to the surrounding area decreases rapidly. This can better protect the organs at risk (OARs) [1,2,3,4,5]. As many patients are already in the advanced stage of cervical cancer after their first consultation and have lost the opportunity for operation, the mortality and incidence rates of cervical cancer are increasing year by year [6,7]. Depending on the specific condition of these patients, in addition to the commonly used first-line treatment, including surgery, radiotherapy, and chemotherapy, targeted therapy and tumor immunotherapy are also research hotspots in recent years. However, few second-line treatment options are available in the event of disease progression, and improvements in overall survival or disease-free progression rate are not sufficiently pronounced [8,9]. HDR BT is widely accepted as an effective treatment method [10]. However, because of concerns, including the steep dose gradient and fluctuating dose absorption in the tumor and normal tissues, HDR BT is still facing a number of dosimetric challenges [11,12]. Consequently, obtaining the dose distribution around the brachytherapy source is crucial from a clinical standpoint.

The International Commission on Radiation Units and Measurement (ICRU) report 89 recommends 60Co and 192Ir as HDR BT sources [13]. In the early days, because the geometry of the 60Co source was larger compared to that of the 192Ir source, doctors preferred the use 192Ir as the primary HDR radiation source in clinical practice. Nevertheless, 60Co has a long half-life and only needs to be replaced about every 5 years. While 192Ir has a half-life of 74 days, it has obvious advantages in terms of human resources, logistics, and economics. The 60Co source is certainly more cost-effective for developing countries [14,15,16,17,18]. As the technology is being updated and developed continually, there is growing concern about the application of the miniaturized 60Co source, so the physical properties and dosimetric parameters of 60Co have been continuously studied. One important point is that the energy of 60Co (1.25 MeV) is higher than that of 192Ir (0.38 MeV), and people suspect that the use of 60Co sources will result in reduced efficacy, increased toxic reactions, and a series of issues, such as determining how to carry out radiation protection.

In this study, we summarize papers on the dose distribution and clinical applications of the HDR 192Ir and 60Co radiation sources in brachytherapy for cervical cancer published by other medical centers in recent years. The two main devices considered for equipment are 192Ir (Nucletron microSelectron HDR-version 2 (mHDR-V2)) and 60Co (Eckert & Ziegler BEBIG Corporation (Co0.A86)), which are the devices used by our center. We compare our findings with other research results to analyze whether there is a significant difference in treatment between 192Ir and 60Co and provide a reference for clinical workers.

## 2. Dosimetric Parameters

**Figure 1:**
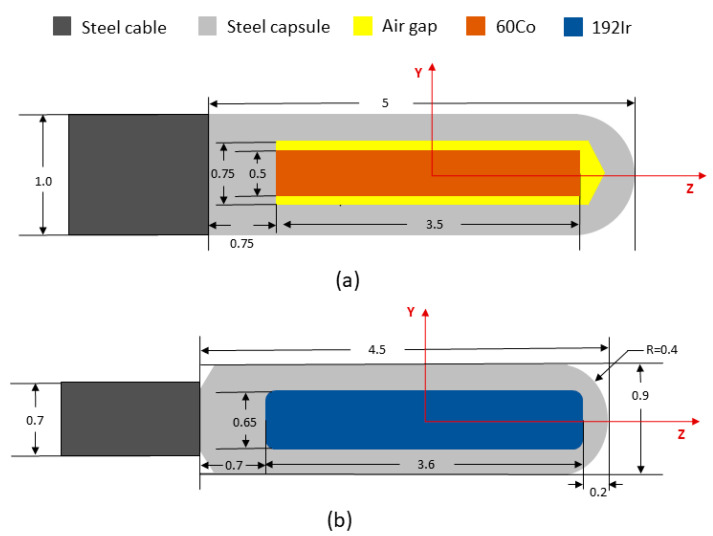
Reference coordinate system and schematic diagram of the model for 60Co and 192Ir. Dimensions are in millimeters. (**a**) E&Z Bebig HDR 60Co model Co0.A86 source; (**b**) Nucletron HDR 192Ir model mHDR-v2r.

Many medical HDR BT resources are currently available. All aspects of the source’s design, composition, and parameters are provided by the manufacturer. The American Association of Physicists in Medicine (AAPM) Task Group 43 (TG-43) recommends that the dose distribution of brachytherapy sources is acquired by experimentation or by using Monte Carlo algorithms. Monte Carlo (MC) algorithms are considered the “gold standard” for radiation therapy dose calculation. The use of MC allows for accurate dose prediction, especially in highly complex and heterogeneous environments, such as human tissue. It is vital to determine the dose characteristics of brachytherapy sources. It is necessary to conduct a dosimetric analysis based on the TG-43 report in order to validate the input simulation results. Previously, Granero et al. obtained 60Co and 192Ir source dose rate distribution data for modern brachytherapy using Monte Carlo methods [15]. Several other researchers have also published their dosimetric parameters for 60Co, which are consistent with published data [19,20,21]. For single 192Ir and 60Co sources, by comparing anisotropy and the radial dose function, it was found that the actual dose deposition of 60Co has no obvious advantages or disadvantages compared with 192Ir [22,23]. A report from AAPM and ESTRO was published in 2012. This recommends dose characteristics for sources of photon-emitting brachytherapy with an average emitted energy above 50 keV, which made it possible to establish the source coordinate system for 192Ir and 60Co (Figure 1).

**Figure 2:**
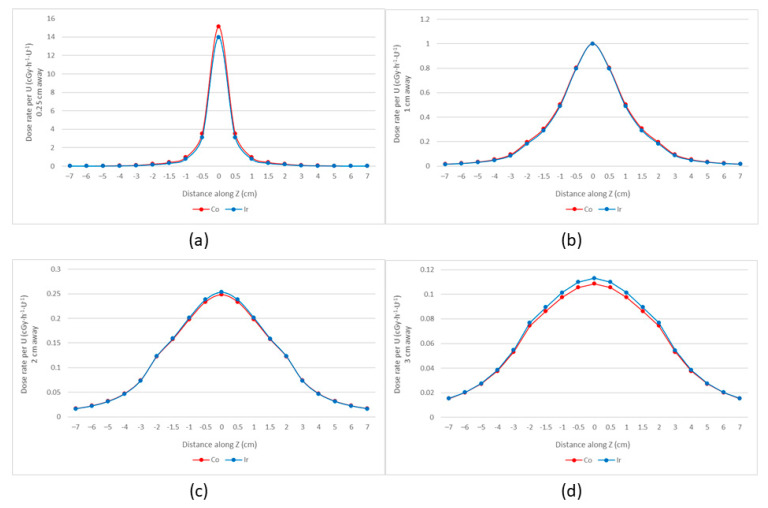
Dose rates of 192Ir (mHDR−V2) and 60Co (Co0.A86) (normalized at y = 1 cm). (**a**) y = 0.25 cm; (**b**) y = 1 cm; (**c**) y = 2 cm; (**d**) y = 3 cm. The 60Co source produced higher dose rates at a distance from the source of less than 1 cm, while the 192Ir source produced higher dose rates at a distance from the source of more than 1 cm.

**Figure 3:**
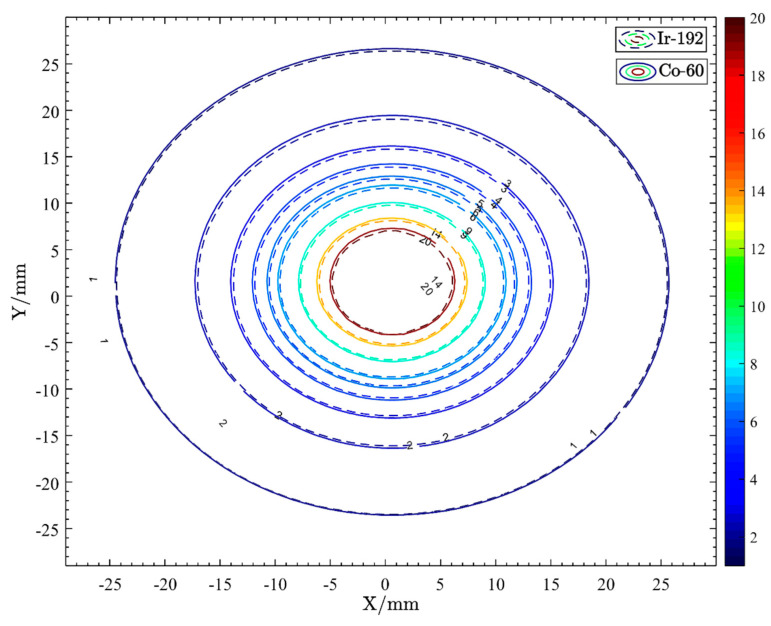
Isodose line of single points of 192Ir and 60Co.

We normalized the recommended dose rate parameters for both sources at the dose reference point (1 cm) and reduced them to the coordinate system (Figure 2). The figures produced show that 60Co and 192Ir have similar dose distribution trends with 60Co producing higher dose rates at a distance from the source of less than 1 cm and 192Ir producing higher dose rates at a distance from the source of more than 1 cm. This indicates that the dose gradient of 60Co is greater than that of 192Ir, as also confirmed by Safigholi et al. [24]. The 60Co source has a faster dose rate decrease and may provide better protection to surrounding OARs. For the majority of points more than 10 mm away from the source center, even if the source step is changed, Farhood et al. concluded that the dose distributions of both sources are stable and, therefore, can be substituted for each other [25]. Dayyani et al. found slightly larger hot spots within the clinical target volume (CTV) when using the 60Co source, possibly due to the steeper dose gradient of 60Co. The dose values for 60Co were higher than those of 192Ir in this area but lower outside when the same dose was delivered to the CTV perimeter (4 cm from the source) [26]. This matched the features of the dataset. Meanwhile, we used the Oncentra treatment planning system (TPS) to place a single source in the plans of 192Ir and 60Co, respectively, and normalized the isodose line at the dose reference point (θ = 90°, y = 1 cm). As shown in Figure 3, within a distance of less than 25 mm from the source, it is apparent that there is little difference in the dose distribution of the two sources.

At most corresponding points, the dose distributions of the two sources are comparable given the same step length within a certain distance from the source. However, in addition to the size and distance from the radiation source, tissue absorption and scattering also affect the dose distribution surrounding the radiation source. The dose distribution of a radioactive source generally follows the inverse square law close to the source. But at higher distances, both absorption and scattering processes affect the dose distribution. Absorption by human tissue results in a reduction in the actual dose, while scattering increases the dose. In most places within 10 cm from the source’s center, the reference value provided by the data set is consistent with the actual dose distribution of the two sources. The dose inhomogeneity of the 60Co source is greater than that of the 192Ir source at 15 cm from the source [25]. The difference between 60Co and the published datasets in the area above 20 cm can be as high as 43.16% [19]. The 192Ir source has a slightly higher dose value. This is due to the fact that the TG-43U1 form’s dosimetry calculation assumes that the patient is made out of water with a uniform density and ignores the heterogeneity of tissues in different parts of the human body. For example, the dose distribution of the radiation source is slightly different between bone, adipose tissue, and tumor tissue. Therefore, in the actual application process, the researchers’ detection of a radiation source will not have the same dosimetric characteristics as the data set. In this case, treating the patient according to the value calculated by TPS may lead to the tumor receiving an insufficient dose. Having too high or too low of a dose may lead to tumor recurrence or other side effects, thereby reducing the treatment efficacy [27,28].

## 3. Two-Dimensional Brachytherapy

For a long period of time, treatment of cervical cancer had to rely on X-ray-guided two-dimensional(2D) HDR BT. The ICRU 38 report specified the prescription reference point and the bladder and rectal reference points as the method of dose assessment. The current traditional 2D HDR BT method uses point A as the dose reference point (point A, i.e., 2 cm vertically upward of the vaginal vault, intersects with the uterus 2 cm left and right outside the central axis, anatomically equivalent to the intersection of the uterine artery and ureter). Point B is located at the point extending horizontally outward 3 cm from point A. According to the Manchester system of the source distribution, each position should be determined with the same dwell time to obtain the classical pear-shaped distribution curve. Dose control for tumors or OARs is not accurate, because target areas and OARs are not outlined. However, the report specifies dose reference points for the bladder and rectum with an aim of minimizing the exposure dose to OARs.

**Figure 4:**
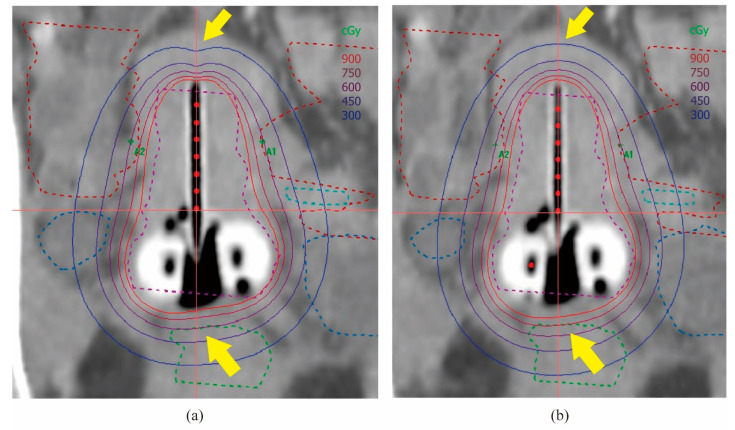
(2D HDR BT) Distribution of isodose lines in the transseptal canal’s coronal plane. (**a**) 192Ir; (**b**) 60Co. As indicated by the arrows, 60Co was more prominently distributed in the cephalocaudal direction and provided a higher dose in the rectum.

We made 2D HDR BT plans of 192Ir and 60Co sources on the same patient’s CT. The reference points of the rectum and bladder were defined according to ICRU 38, and the prescribed dose at point A was 6 Gy with no more than 60–70% of the dose at point A being delivered to the rectum and bladder. The dose distribution in the coronal plane of the uterine cavity is shown in Figure 4. The dose distribution of 60Co in the cephalocaudal direction is more prominent, while that in the horizontal direction is slightly more adducted than 192Ir, and 60Co at the rectum provides a higher dose [29]. Data from Palmer et al. showed no statistically significant difference in high-risk clinical target volume (HRCTV) D90 (dose to 90% volume) and also no significant difference in bladder reference points between the two 2D HDR BT treatment plans of 60Co and 192Ir; however, the rectal reference point dose was significantly higher for 60Co compared with that for 192Ir (+3.3% (*p* < 0.01) and +2.2% (*p* = 0.03), respectively) [29]. Park et al. made 2D HDR BT treatment plans using 60Co (BEBIG) and 192Ir (microselectron) and found that, compared with 192Ir, the dose distribution of the 60Co source was 5.6 ± 0.23% lower at point B, 0.83 ± 1.48% higher at the rectal reference point, and 1.14 ± 0.61% lower at the bladder reference point [23]. We consider that this may be due to the inherent physical properties of the two sources, which can be continuously optimized to narrow the gap between them.

In terms of clinical care, 174 cervical cancer patients who received brachytherapy were included in the study by Ulinskas et al. The 5-, 10-, and 15-year survival rates of the 60Co group were 35.4%, 26.9%, and 22.5%, and disease-free survival rates were 32.0%, 25.1%, and 21.4%, respectively [30]. A retrospective study by Pesee et al. of 141 cervical cancer patients treated with 60Co brachytherapy showed an overall 5-year survival rate of 63.3% and rates of 100%, 80.3%, 100%, and 54.8% for stage IB, IIB, IIIA, and IIIB cancers, respectively [31]. The incidence of grade 2 radiation proctitis was slightly higher in patients treated with HDR BT with the 850 cGy/fraction [32]. Tantivatana et al. conducted a 5-year follow-up study of patients treated with 2D HDR BT with 192Ir or 60Co sources and found no noticeable differences in the overall survival (77% vs. 81.9%), disease-free survival (73.1% vs. 74.7%), and grade 3 and 4 complications (4.7% vs. 3.4%) [33]. Rakhsha et al. studied 154 patients who received 60Co treatment for cervical cancer. The median follow-up time was 38 months. The rectal and bladder toxicity rate was 33.7%, and the 3-year DFS rates for stage I, II, III, and IVA cancer were 85.7%, 70.7%, 41%, and 16.6%, respectively [34]. In the prospective trial reported by Ntekim et al., two patients (3%) with cervical cancer treated with 60Co brachytherapy had grade 3 gastrointestinal toxicity. All other patients had ≤grade 2 toxicity, and the overall acute gastrointestinal and genitourinary toxicity levels were low and comparable to the toxicity reported for the 192Ir HDR source [35]. Tanaka et al. used the 192Ir HDR source for brachytherapy in 135 patients with cervical cancer (22 in stage I, 49 in stage II, 56 in stage III, and 8 in stage IVA) with 5-year survival rates of 90%, 78%, 53%, and 33% in stages I, II, III, and IVA, respectively [36]. Song et al. conducted a retrospective analysis of 76 patients treated with 60 Co. The overall survival rates and five-year progression-free survival were found to be 69.3% and 63.7%, respectively [37]. Thakur et al. performed a retrospective analysis of all patients treated with Ir HDR BT three times at 7 Gy in group A and twice at 9 Gy in group B. The two-year local control rates were 88.5% and 91.5%, disease-free survival rates were 85.9% and 82.6%, and overall survival rates were 95.7% and 100% in groups A and B, respectively [38]. According to Hochreiter et al., the 3-year overall survival rate, disease-free survival rate, and pelvic recurrence-free survival rate of 111 patients with endometrial cancer after 192Ir brachytherapy were 89.6%, 90.1%, and 92.8%, respectively [39]. From the clinical trials reported so far, the use of both 192Ir and 60Co in HDR BT of cervical cancer patients can reduce mortality and improve survival. There are no particular advantages for 192Ir in terms of toxic reactions and survival, and there is no discernible difference between the two radiation sources’ therapeutic effects. However, there is uncertainty in the locations of the applicator, rectum, and bladder due to the presence of patient handling, bowel movements, and other factors. There is a possibility that the dose received at the actual reference point and in the rectum, bladder, and other organs may not coincide with the results calculated by TPS for 2D HDR BT [40,41,42,43]. Therefore, it is not a good predictor of patient recovery and the probability of normal tissue complications [44,45,46,47].

## 4. Three-Dimensional Intracavity Brachytherapy

With the extensive development of brachytherapy for cervical cancer, people are gradually paying attention to the limitations of traditional 2D HDR BT based on X-rays for cervical cancer: The individualized distribution of tumors is not taken into account, and the location, size, and degree of invasion of surrounding tissues cannot be accurately displayed. The dose distribution of the whole treatment plan cannot be adjusted and optimized. Thus, the clinical local control rate, toxicity response, and survival rate cannot be improved [48,49,50,51]. Recent years have seen remarkable advances in intensity-modulated radiation technology (IMRT), and image-guided three-dimensional (3D) HDR BT has been applied and promoted. Image-guided 3D BT is also based on individualized anatomical structures. By continuously optimizing the brachytherapy planning system, an optimal treatment plan can be determined, which can significantly improve the accuracy of the CTV and OARs dose assessment and provide adequate dose coverage for CTV without excessive doses of OARs. Therefore, 3D HDR BT technology has clear advantages over 2D technology for clinical treatment [52,53,54,55,56,57].

**Figure 5:**
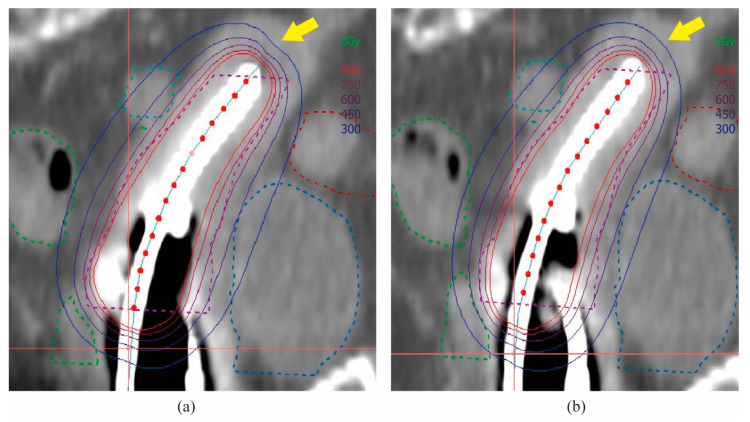
(3D HDR ICBT) Isodose line distribution in the sagittal plane of the transseptal canal. (**a**) 192Ir; (**b**) 60Co. As indicated by the arrows, 60Co was more prominently distributed in the cephalocaudal direction.

We used CT images of the same patient to produce 3D HDR BT plans for both 192Ir and 60Co sources. The sagittal dose distribution is shown (Figure 5). Compared with 192Ir, the dose distribution of 60Co is more prominent in the cephalocaudal direction, which is a similar result to that obtained by Shukla [58] and Palmer [29]. There are slight variations in the dose distribution due to the physical properties of these isotopes, which vary naturally. With treatment plan optimization techniques, the differences in the CTV dose can be reduced so that they are essentially the same in terms of actual clinical treatment outcomes. At a prescribed dose of 6 Gy of CTV D90, the difference in dose between 60Co and 192Ir at D1cc and D2cc of OARs is less than 10 cGy. At the bladder and rectum, there is a small increase in the dose of 60Co compared to 192Ir. This part of the difference can be agreed upon for actual clinical treatment through continuous optimization of the physical plan without affecting the final treatment [59].

Sinnatamby et al. analyzed brachytherapy treatment plans for 27 patients and found minimal differences in the dose distribution when using 60Co or 192Ir as the radiation source [60]. In the study by Gujar et al. on 60Co, the mean HRCTV D90 was 21.4 ± 0.69 Gy in all patients, and the mean D2cc was 15.9 ± 0.58 Gy, 11.5 ± 0.91 Gy, and 4.1 ± 1.52 Gy in the bladder, rectum and sigmoid colon, respectively [61]. Tormo Ferrero et al. analyzed 3D HDR BT plans with 192Ir and 60Co sources and compared the dose differences, showing that, other from the dose percentage difference at point “1” (5 mm tissue depth from the top of the vaginal cylinder), there were no statistically significant changes in dose percentages for the rectum., bladder, and sigmoid D2cc or for point 0 (top surface of the vaginal cylinder) [62]. Richter J et al. reported that 60Co and 192Ir sources of the same shape and structure showed almost identical dose distributions, with 60Co doses in the rectum being 0.8% lower than those of 192Ir sources [22]. In the study by Mobit et al., it was found that, with a 2.2 cm vaginal cylinder, the dose at 90% of the planned target volume (V90%) was 97% and 98% of the prescribed dose for 60Co versus 192Ir, 20.2% and 17.3% for V150%, and 3.9% and 3.5% for V200%, respectively. With a 2.6 cm vaginal cylinder, the DVH for 60Co versus that for 192Ir differed slightly. With the 3.0 cm vaginal cylinder, for 60Co and 192Ir, V90% was 98% and 99%, V150% was 11.8% and 10.7%, and V200% was 3.1% and 2.7%, respectively [63]. The above results show almost no significant difference in the physical plans of the two radiation sources. Therefore, we believe that there is no major difference in the selection of 60Co versus 192Ir in 3D HDR BT.

**Figure 6:**
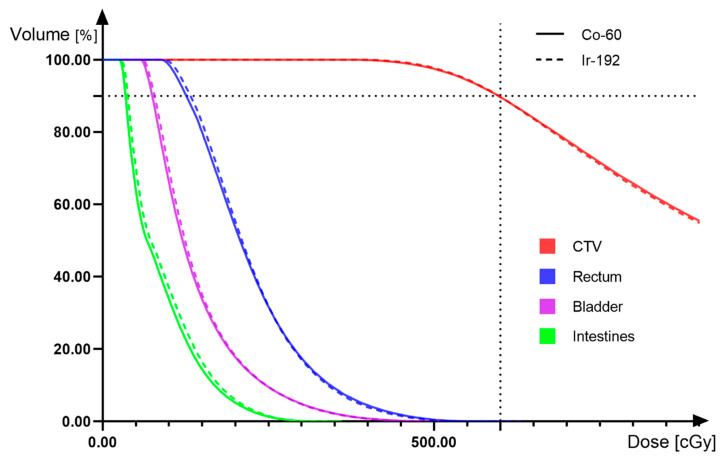
(3D HDR ICBT) Dose-volume histogram comparing 192Ir and 60Co.

In our study, the dose-volume histogram (DVH) of the 3D HDR BT plans for 192Ir and 60Co are shown in Figure 6, and the curves of the two are basically coincident. For OARs, the dose of 60Co is slightly lower than that of 192Ir, so we speculate that the use of 60Co may have a better protective effect. This is slightly different from the results of Suresh et al., who found that the average rectal dose of 60Co was higher than that of 192Ir, but the average dose of 60Co was lower than that of 192Ir for the bladder and sigmoid. They mentioned that, compared with the 192Ir radioisotope, the absorption, attenuation, and scattering effects of the 60Co radioisotope are correspondingly reduced due to the higher average gamma energy. When using 60Co, reductions in these factors will lead to a lesser dose being received by the target tissue, yet high doses being transmitted to distant tissues (OARs) [64]. We found that, on the one hand, these differences originate from differences in the intrinsic physical properties of the 60Co and Ir192 sources, because 60Co has a higher gamma capability, and its internal self-absorption is less than that of 192Ir. On the other hand, different physicists give different weights to the dose optimization of CTV and OARs in radiotherapy plans, resulting in different dose results. In actual clinical applications, in order to ensure the prescribed dose of CTV is received and to simultaneously eliminate the dose difference brought about by the choice of the radiation source or the subjective operation of the physicist, adjusting the step radiation source’s dwell time will optimize the dose distribution, which is the advantage of brachytherapy [65].

In terms of clinical treatment, Atasever et al. performed a retrospective analysis of 50 patients with stage IA-IIIC2 cervical cancer treated with 3D HDR ICBT using 192Ir. The results were 76% complete remission, 4% partial remission, 12% presence of local recurrence, and one-year and five-year survival rates of 88% and 67.5%, respectively [66]. Wang et al. reported a retrospective analysis of 373 patients with stage IIB cervical cancer undergoing HDR ICBT using 192Ir. The disease-free survival, three-year overall survival, and local control rates were 82.2%, 87.5%, and 92.5%, respectively [67]. In a study by Kusada et al. on 192Ir HDR ICBT, the local control 2-year rates of pelvic control and progression-free survival were 85%, 83%, and 75%, respectively [68]. At present, there is a lack of clinical trials for 3D HDR ICBT in cervical cancer patients using 60Co alone, and by recording clinical outcomes such as survival, toxicity, or local recurrence, we will continue to pay close attention to progress in this regard.

## 5. 3-Dimensional Intracavity-Interstitial Brachytherapy

Since the tumor will invade normal tissues in all directions during the process of growth, especially for some large tumors, the main body of which may be far from the cervix, brachytherapy will not provide a suitable dose for irradiation if it relies on the uterine cavity tube alone, so intracavity-interstitial brachytherapy (IC-ISBT) has been further developed and promoted. External irradiation combined with simple intracavitary brachytherapy has been proven to be an incredibly efficient method for the treatment of cervical cancer in its early stages, but still has limitations when used for intermediate and advanced cervical cancer [5,69,70]. HDR IC-ISBT can place the needles along the tumor morphology and combine with the intracavitary applicator, guaranteeing that a large dose is transmitted to the center of the cervix while providing the tumor target area with highly conformal coverage. At the same time, through technical optimization, the dose given to the OARs does not exceed the upper limit, so it has a better local control rate for advanced large-volume cervical cancer [71,72,73,74,75,76]. Previously, the 60Co source was not suitable for use with interstitial techniques due to its large size, and current technology has improved. It has been shown that there is no significant difference in dose distribution in CTV and OARs for HDR IC-ISBT treatment plans using 60Co or 192Ir sources [22]. With the development of HDR IC-ISBT for cervical cancer, more patients with advanced cervical cancer have been effectively controlled [53,77].

**Figure 7:**
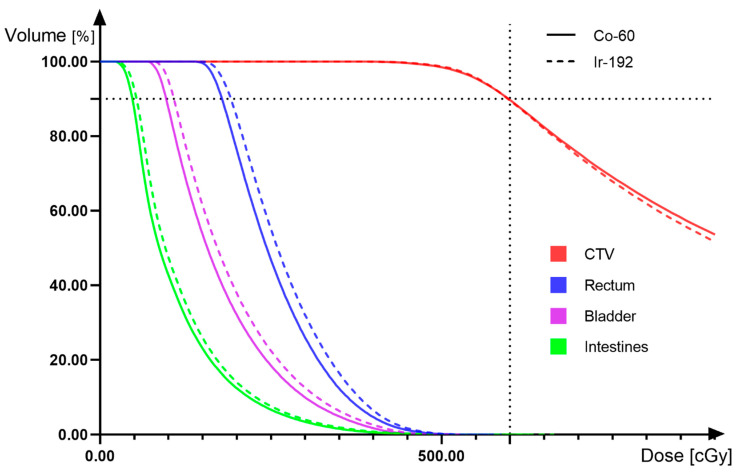
(3D HDR IC-ISBT) Dose-volume histogram comparing 192Ir and 60Co.

**Figure 8:**
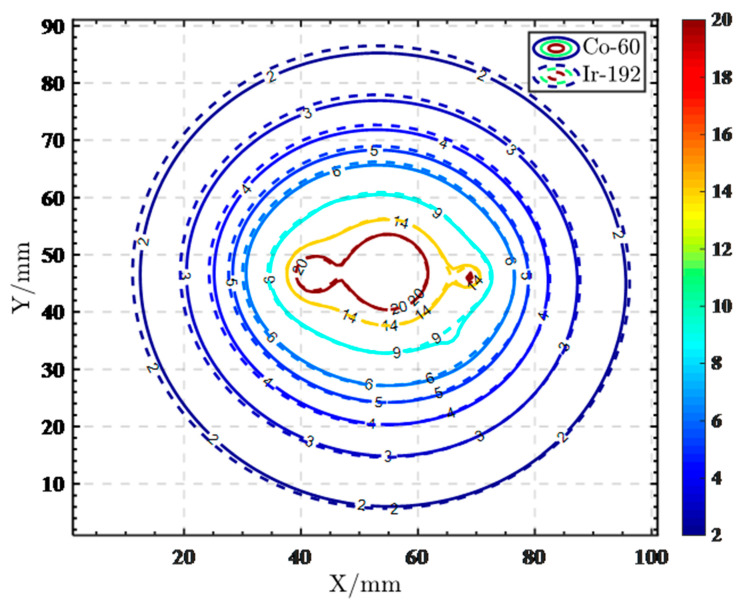
(3D HDR IC-ISBT) Cross-sectional isodose line distribution of 192Ir and 60Co.

We made 60Co and 192Ir HDR IC-ISBT plans for the CTs of 30 patients, respectively, and in accordance with the ICRU 89 document, delineated the target volume on the CT and derived the dose data for comparison. The CTV D90 of 60Co and 192Ir plans were both 6Gy. The average physical dose values of OARs (bladder, rectum, intestines) D_2CC_ were as follows (Table 1). The paired *t*-test results showed a statistical difference in dose between the two radiation source physics plans at the OARs, and the mean dose values for OARs of 60Co HDR IC-ISBT plans were slightly lower than those for 192Ir. We randomly selected two treatment plans for one patient with two different radiation sources and derived the DVH plot (Figure 7), which shows that the CTV curves largely overlap. In OARs, the dose value of 60Co was lower than that of 192Ir, and it is possible that 60Co has a better protective effect on OARs. The cross-sectional dose distribution (Figure 8) shows that the HDR IC-ISBT plans produced using 60Co and 192Ir sources were also largely consistent in terms of dose distribution after optimization by the physicist. Our results are similar to those of Mourougan et al. who performed a study on the use of HDR ISBT for breast cancer treatment using 60Co and 192Ir. The results showed that the volume difference between 192Ir and 60Co contained in the reference isodose line was 2.34% (*p* < 0.05). There were 2.5% and 1.2% differences in V150% and V200%, respectively, and the results were found to be statistically significant. However, Mourougan pointed out that the physical differences shown here are not clinically significant, and the actual 60Co dose distribution is essentially the same as that of 192Ir [78].

In terms of clinical treatment, Mohan Kumar et al. used 60Co for HDR IC-ISBT, and the results showed that the median EQD_2_ of the 2 cm^3^ bladder, rectum, sigmoid, and D90 CTV_HR_ were 70 Gy (53–75 Gy), 64 Gy (51–71 Gy), 48 Gy (44–72 Gy), and 77 Gy (70–86 Gy), respectively, while the 2-year local control rate was 87.14% [79]. Tiwari et al. reported significantly better local control (90.4% vs. 76.2%) and DFS (78.8% vs. 57.1%) in patients in the IC-ISBT group compared to those in the ICBT group with no discernible difference in the mean dose for the bladder and sigmoid 2cc but a statistically lower dose for the rectum 2cc [80]. A study involving 315 patients with locally advanced cervical cancer who received HDR ISBT of 192Ir was conducted by Fallon et al. More than half of the patients (51%) were treated with 3D HDR ICBT, and it was discovered that the overall local control (2D and 3D groups) was 87% at 10 years. The 2D and 3D results were not reported separately; however, the entire trial continues to demonstrate that HDR ISBT is a secure and efficient treatment [81]. Murakami et al. reported that, in their study, the two-year overall survival rate, local control rate, and progression-free survival rate were 81.6%, 80.2%, and 54.4%, respectively, for patients treated with 192Ir HDR ISBT [82]. The 5-year follow-up of the entire cohort of patients treated with 192Ir HDR ISBT by le Guyader et al. showed local recurrence-free survival (LFS) in 85% of patients, lymph node recurrence-free survival (NFS) in 83% of patients, metastasis-free survival (MFS) in 70% of patients, progression-free survival (PFS) in 61% of patients, and an overall survival (OS) rate of 75% [83]. In comparison to the findings of previous studies, the results for the dosimetric parameters, local control, and toxicity of cervical cancer patients treated with HDR ISBT using 60 Co were found to be basically consistent with those of 192Ir.

## 6. Conclusions

By retrieving the relevant domestic and foreign literature published over the past few years, we comparatively analyzed the differences in dosimetric and clinical outcomes between treatment with 60Co and 192lr. The results showed that, although the initial physical properties differ, there are no clear differences in dosimetric parameters in the brachytherapy planning system after optimization. In terms of actual clinical effects, small dose differences will not cause significant differences in the toxicity, disease-free survival, local recurrence rate, or survival rate of patients. Compared with 192Ir, 60Co has no obvious advantages or disadvantages as an HDR brachytherapy source. It is safe and feasible to use 60Co for treatment. In particular, 60Co has a long half-life and is replaced about every 5 years, which will save a lot of manpower and reduce economic costs for developing countries. We recommend the use of 60Co as a high-dose-rate brachytherapy source in clinical practice, especially in developing countries.

## Figures and Tables

**Table 1:** Mean physical doses for CTV and OARs $\left(\bar{X}+S\right)$.

	CTV D90/Gy	Bladder D2cc/Gy	Rectum D2cc/Gy	Intestines D2cc/Gy
**Co**	6	4.70 ± 0.29	4.41 ± 0.68	3.57 ± 1.12
**Ir**	6	4.81 ± 0.28	4.49 ± 0.68	3.66 ± 1.13
***p* value**		*p* < 0.05	*p* < 0.05	*p* < 0.05

## Data Availability

All data generated or analyzed during this study are included in this published article.

## References

[1] Viswanathan A.N., Thomadsen B., American Brachytherapy Society Cervical Cancer Recommendations Committee, American Brachytherapy Society (2012). American Brachytherapy Society consensus guidelines for locally advanced carcinoma of the cervix. Part I: General principles. Brachytherapy.

[2] Viswanathan A.N., Beriwal S., De Los Santos J.F., Demanes D.J., Gaffney D., Hansen J., Jones E., Kirisits C., Thomadsen B., Erickson B. (2012). American Brachytherapy Society consensus guidelines for locally advanced carcinoma of the cervix. Part II: High-dose-rate brachytherapy. Brachytherapy.

[3] Milickovic N., Tselis N., Karagiannis E., Ferentinos K., Zamboglou N. (2017). Iridium-Knife: Another knife in radiation Oncology. Brachytherapy.

[4] Gill B.S., Lin J.F., Krivak T.C., Sukumvanich P., Laskey R.A., Ross M.S., Lesnock J.L., Beriwal S. (2014). National Cancer Data Base analysis of radiation therapy consolidation modality for cervical cancer: The impact of new technological advancements. Int. J. Radiat. Oncol. Biol. Phys..

[5] Han K., Milosevic M., Fyles A., Pintilie M., Viswanathan A.N. (2013). Trends in the utilization of brachytherapy in cervical cancer in the United States. Int. J. Radiat. Oncol. Biol. Phys..

[6] Xia C., Dong X., Li H., Cao M., Sun D., He S., Yang F., Yan X., Zhang S., Li N. (2022). Cancer statistics in China and United States, 2022: Profiles, trends, and determinants. Chin. Med. J..

[7] Siegel R.L., Miller K.D., Fuchs H.E., Jemal A. (2022). Cancer statistics, 2022. CA Cancer J. Clin..

[8] Pfaendler K.S., Tewari K.S. (2016). Changing paradigms in the systemic treatment of advanced cervical cancer. Am. J. Obstet. Gynecol..

[9] McLachlan J., Boussios S., Okines A., Glaessgen D., Bodlar S., Kalaitzaki R., Taylor A., Lalondrelle S., Gore M., Kaye S. (2017). The Impact of Systemic Therapy Beyond First-line Treatment for Advanced Cervical Cancer. Clin. Oncol..

[10] Berman T.A., Schiller J.T. (2017). Human papillomavirus in cervical cancer and oropharyngeal cancer: One cause, two diseases. Cancer.

[11] Musunuru H.B., Pifer P.M., Mohindra P., Albuquerque K., Beriwal S. (2022). Advances in management of locally advanced cervical cancer. Indian J. Med. Res..

[12] Chargari C., Peignaux K., Escande A., Renard S., Lafond C., Petit A., Lam Cham Kee D., Durdux C., Haie-Meder C. (2022). Radiotherapy of cervical cancer. Cancer Radiother..

[13] International Commission on Radiation Units and Measurements (2016). ICRU Report 89: Prescribing, Recording, and Reporting Brachytherapy for Cancer of the Cervix.

[14] Strohmaier S., Zwierzchowski G. (2011). Comparison of (60)Co and (192)Ir sources in HDR brachytherapy. J. Contemp. Brachytherapy.

[15] Granero D., Perez-Calatayud J., Ballester F. (2007). Technical note: Dosimetric study of a new Co-60 source used in brachytherapy. Med. Phys..

[16] Ott O.J., Lotter M., Sauer R., Strnad V. (2007). Accelerated partial-breast irradiation with interstitial implants: The Clinical relevance of the calculation of skin doses. Strahlenther. Onkol..

[17] Salminen E.K., Kiel K., Ibbott G.S., Joiner M.C., Rosenblatt E., Zubizarreta E., Wondergem J., Meghzifene A. (2011). International Conference on Advances in Radiation Oncology (ICARO): Outcomes of an IAEA meeting. Radiat. Oncol..

[18] Mailhot Vega R.B., Barbee D., Talcott W., Duckworth T., Shah B.A., Ishaq O.F., Small C., Yeung A.R., Perez C.A., Schiff P.B. (2018). Cost in perspective: Direct assessment of American market acceptability of Co-60 in Gynecol.ogic high-dose-rate brachytherapy and contrast with experience abroad. J. Contemp. Brachytherapy.

[19] Badry H., Oufni L., Ouabi H., Hirayama H. (2018). A Monte Carlo investigation of the dose distribution for (60)Co high dose rate brachytherapy source in water and in different media. Appl. Radiat. Isot..

[20] Reddy B.R., Chamberland M.J.P., Ravikumar M., Varatharaj C. (2019). Measurements and Monte Carlo calculation of radial dose and anisotropy functions of BEBIG (60)Co high-dose-rate brachytherapy source in a bounded water phantom. J. Contemp. Brachytherapy.

[21] Mozaffari A., Ghorbani M. (2019). Determination of TG-43 Dosimetric Parameters for Photon Emitting Brachytherapy Sources. J. Biomed. Phys. Eng..

[22] Richter J., Baier K., Flentje M. (2008). Comparison of 60cobalt and 192iridium sources in high dose rate afterloading brachytherapy. Strahlenther. Onkol..

[23] Park D.W., Kim Y.S., Park S.H., Choi E.K., Ahn S.D., Lee S.W., Song S.Y., Kim J.H. (2009). A comparison of dose distributions of HDR intracavitary brachytherapy using different sources and treatment planning systems. Appl. Radiat. Isot..

[24] Safigholi H., Meigooni A.S., Song W.Y. (2017). Comparison of (192) Ir, (169) Yb, and (60) Co high-dose rate brachytherapy sources for skin cancer treatment. Med. Phys..

[25] Farhood B., Ghorbani M. (2017). Assessment of dose uniformity around high dose rate (192)Ir and (60)Co stepping sources. Radiol. Phys. Technol..

[26] Dayyani M., Hoseinian-Azghadi E., Miri-Hakimabad H., Rafat-Motavalli L., Abdollahi S., Mohammadi N. (2021). Radiobiological comparison between Cobalt-60 and Iridium-192 high-dose-rate brachytherapy sources: Part I-cervical cancer. Med. Phys..

[27] Huang R., Zhou Y., Hu S., Ren G., Cui F., Zhou P.K. (2019). Radiotherapy Exposure in Cancer Patients and Subsequent Risk of Stroke: A Systematic Review and Meta-Analysis. Front. Neurol..

[28] Mu H., Sun J., Li L., Yin J., Hu N., Zhao W., Ding D., Yi L. (2018). Ionizing radiation exposure: Hazards, prevention, and biomarker screening. Environ. Sci. Pollut. Res. Int..

[29] Palmer A., Hayman O., Muscat S. (2012). Treatment planning study of the 3D dosimetric differences between Co-60 and Ir-192 sources in high dose rate (HDR) brachytherapy for cervix cancer. J. Contemp. Brachytherapy.

[30] Ulinskas K., Janulionis E., Valuckas K.P., Samerdokiene V., Atkocius V., Rivard M.J. (2016). Long-term results for Stage IIIB cervical cancer patients receiving external beam radiotherapy combined with either. HDR (252)Cf or HDR (60)Co intracavitary brachytherapy. Brachytherapy.

[31] Pesee M., Krusun S., Padoongcharoen P. (2010). High dose rate cobalt-60 afterloading intracavitary therapy for cervical carcinoma in Srinagarind hospital—Analysis of survival. Asian Pac. J. Cancer Prev..

[32] Pesee M., Krusun S., Padoongcharoen P. (2010). High dose rate cobalt-60 afterloading intracavitary therapy of uterine cervical carcinomas in Srinagarind hospital—Analysis of complications. Asian Pac. J. Cancer Prev..

[33] Tantivatana T., Rongsriyam K. (2018). Treatment outcomes of high-dose-rate intracavitary brachytherapy for cervical cancer: A comparison of Ir-192 versus Co-60 sources. J. Gynecol. Oncol..

[34] Rakhsha A., Kashi A.S.Y., Hoseini S.M. (2015). Evaluation of Survival and Treatment Toxicity With High-Dose-Rate Brachytherapy with Cobalt 60 in Carcinoma of Cervix. Iran. J. Cancer Prev..

[35] Ntekim A., Adenipekun A., Akinlade B., Campbell O. (2010). High Dose Rate Brachytherapy in the Treatment of cervical cancer: Preliminary experience with cobalt 60 Radionuclide source-A Prospective Study. Clin. Med. Insights Oncol..

[36] Tanaka E., Suzuki O., Oh R.J., Takeda T., Teshima T., Inoue T., Inoue T. (2006). Intracavitary brachytherapy for carcinoma of the uterine cervix—Comparison of HDR (Ir-192) and MDR (Cs-137). Radiat. Med..

[37] Song J., Alyamani N., Bhattacharya G., Le T., E C., Samant R. (2020). The Impact of High-Dose-Rate Brachytherapy: Measuring Clin.ical Outcomes in the Primary Treatment of Cervical Cancer. Adv. Radiat. Oncol..

[38] Thakur P., Dogra E., Gupta M., Negi R.R., Fotedar V., Thakur S., Sharma C. (2019). Comparison of iso-effective and cost-effective high-dose-rate brachytherapy treatment schedules in cervical cancer—regional cancer center experience. J. Contemp. Brachytherapy.

[39] Hochreiter A., Kelly J.R., Young M.R., Litkouhi B., Black J.D., Stromberger C., Higgins S., Schwartz P.E., Damast S. (2020). Outcomes and relapse patterns of stage IB grade 2 or 3 endometrial cancer treated with adjuvant vaginal brachytherapy. Int. J. Gynecol. Cancer.

[40] Alecu R., Alecu M. (1999). In-vivo rectal dose measurements with diodes to avoid misadministrations during intracavitary high dose rate brachytherapy for carcinoma of the cervix. Med. Phys..

[41] Huh H., Kim W., Loh J.J., Lee S., Kim C.Y., Lee S., Shin D., Shin D., Cho S., Jang J. (2007). Rectum dose analysis employing a multi-purpose brachytherapy phantom. Jpn. J. Clin. Oncol..

[42] Waldhausl C., Wambersie A., Potter R., Georg D. (2005). In-vivo dosimetry for gynaecological brachytherapy: Physical and Clinical considerations. Radiother. Oncol..

[43] Zaman Z.K., Ung N.M., Malik R.A., Ho G.F., Phua V.C., Jamalludin Z., Baharuldin M.T., Ng K.H. (2014). Comparison of planned and measured rectal dose in-vivo during high dose rate Cobalt-60 brachytherapy of cervical cancer. Phys. Med..

[44] Wang K.L., Yang Y.C., Chao K.S., Wu M.H., Tai H.C., Chen T.C., Huang M.C., Chen J.R., Su T.H., Chen Y.J. (2007). Correlation of traditional point a with anatomic location of uterine artery and ureter in cancer of the uterine cervix. Int. J. Radiat. Oncol. Biol. Phys..

[45] Katz A., Eifel P.J. (2000). Quantification of intracavitary brachytherapy parameters and correlation with outcome in patients with carcinoma of the cervix. Int. J. Radiat. Oncol. Biol. Phys..

[46] Kim R.Y., Pareek P. (2003). Radiography-based treatment planning compared with computed tomography (CT)-based treatment planning for intracavitary brachytherapy in cancer of the cervix: Analysis of dose-volume histograms. Brachytherapy.

[47] Datta N.R., Basu R., Das K.J., Rajasekar D., Pandey C.M., Singh U., Ayyagari S. (2004). Problems and uncertainties with multiple point A’s during multiple high-dose-rate intracavitary brachytherapy in carcinoma of the cervix. Clin. Oncol..

[48] Hashemi F.A., Mansouri S., Aghili M., Esmati E., Babaei M., Saeedian A., Moalej S., Jaberi R. (2021). A comparison between 2D and 3D planning of high-dose-rate vaginal cuff brachytherapy in patients with stage I-II endometrial cancer using cobalt-60. J. Contemp. Brachytherapy.

[49] Suzumura E.A., Gama L.M., Jahn B., Campolina A.G., Carvalho H.A., de Soarez P.C. (2021). Effects of 3D image-guided brachytherapy compared to 2D conventional brachytherapy on clinical outcomes in patients with cervical cancer: A systematic review and meta-analyses. Brachytherapy.

[50] Derks K., Steenhuijsen J.L.G., van den Berg H.A., Houterman S., Cnossen J., van Haaren P., De Jaeger K. (2018). Impact of brachytherapy technique (2D versus 3D) on outcome following radiotherapy of cervical cancer. J. Contemp. Brachytherapy.

[51] Kim Y.J., Kang H.C., Kim Y.S. (2020). Impact of intracavitary brachytherapy technique (2D versus 3D) on outcomes of cervical cancer: A systematic review and meta-analysis. StrahlenTher. Onkol..

[52] Kim H., Beriwal S., Houser C., Huq M.S. (2011). Dosimetric analysis of 3D image-guided HDR brachytherapy planning for the treatment of cervical cancer: Is point A-based dose prescription still valid in image-guided brachytherapy?. Med. Dosim..

[53] Potter R., Georg P., Dimopoulos J.C., Grimm M., Berger D., Nesvacil N., Georg D., Schmid M.P., Reinthaller A., Sturdza A. (2011). Clinical outcome of protocol based image (MRI) guided adaptive brachytherapy combined with 3D conformal radiotherapy with or without chemotherapy in patients with locally advanced cervical cancer. Radiother. Oncol..

[54] Lindegaard J.C., Fokdal L.U., Nielsen S.K., Juul-Christensen J., Tanderup K. (2013). MRI-guided adaptive radiotherapy in locally advanced cervical cancer from a Nordic perspective. Acta Oncol..

[55] Srivastava A., Datta N.R. (2014). Brachytherapy in cancer cervix: Time to move ahead from point A?. World J. Clin. Oncol..

[56] Someya M., Hasegawa T., Tsuchiya T., Kitagawa M., Gocho T., Fukushima Y., Hori M., Miura K., Takada Y., Nakata K. (2020). Retrospective DVH analysis of point A based intracavitary brachytherapy for uterine cervical cancer. J. Radiat. Res..

[57] Bandyopadhyay A., Ghosh A.K., Chhatui B., Das D. (2021). Dosimetric and Clin.ical outcomes of CT based HR-CTV delineation for HDR intracavitary brachytherapy in carcinoma cervix—A retrospective study. Rep. Pract. Oncol. Radiother..

[58] Shukla A.K., Jangid P.K., Rajpurohit V.S., Verma A., Dangayach S.K., Gagrani V., Rathore N.K. (2019). Dosimetric comparison of (60)Co and (192)Ir high dose rate source used in brachytherapy treatment of cervical cancer. J. Cancer Res. Ther..

[59] Moren B., Larsson T., Tedgren A.C. (2021). Optimization in treatment planning of high dose-rate brachytherapy—Review and analysis of mathematical models. Med. Phys..

[60] Sinnatamby M., Kandasamy S., Karunanidhi G., Neelakandan V., Ramapandian S., Kannan M., Sampath E. (2022). Image-Guided Brachytherapy a Comparison Between 192Ir and 60Co Sources in Carcinoma Uterine Cervix. Gulf J. Oncol..

[61] Gurjar O.P., Batra M., Bagdare P., Kaushik S., Tyagi A., Naik A., Bhandari V., Gupta K.L. (2016). Dosimetric analysis of Co-60 source based high dose rate (HDR) brachytherapy: A case series of ten patients with carcinoma of the uterine cervix. Rep. Pract. Oncol. Radiother..

[62] Tormo Ferrero V., Duque Ugarte R., Berenguer Frances M.A., Cardenal Macia R. (2017). Gynecol.ogical brachytherapy for postoperative endometrial cancer: Dosimetric analysis (Ir-192 vs. Co-60). Clin. Transl. Oncol..

[63] Mobit P.N., Nguyen A., Packianathan S., He R., Yang C.C. (2016). Dosimetric comparison of brachytherapy sources for high-dose-rate treatment of endometrial cancer: (192)Ir, (60)Co and an electronic brachytherapy source. Br. J. Radiol..

[64] Yadav S., Singh O.P., Choudhary S., Saroj D.K., Yogi V., Goswami B. (2021). Estimation and comparison of integral dose to target and organs at risk in three-dimensional computed tomography image-based treatment planning of carcinoma uterine cervix with two high-dose-rate brachytherapy sources: (60)Co and (192)Ir. J. Cancer Res. Ther..

[65] Boeck L.D., Belin J., Egyed W. (2014). Dose optimization in high-dose-rate brachytherapy: A literature review of quantitative models from 1990 to 2010. Oper. Res. Health Care.

[66] Atasever Akkas E., Altundag M.B. (2021). Long-term clinical outcome and dosimetric comparison of tandem and ring versus tandem and ovoids intracavitary application in cervical cancer. J. BUON.

[67] Wang W., Meng Q., Hou X., Lian X., Yan J., Sun S., Liu Z., Miao Z., Wang D., Liu X. (2017). Efficacy and toxicity of image-guided intensity-modulated radiation therapy combined with dose-escalated brachytherapy for stage IIB cervical cancer. Oncotarget.

[68] Kusada T., Toita T., Ariga T., Kudaka W., Maemoto H., Makino W., Ishikawa K., Heianna J., Nagai Y., Aoki Y. (2020). Definitive radiotherapy consisting of whole pelvic radiotherapy with no central shielding and CT-based intracavitary brachytherapy for cervical cancer: Feasibility, toxicity, and oncologic outcomes in Japanese patients. Int. J. Clin. Oncol..

[69] Tanderup K., Eifel P.J., Yashar C.M., Potter R., Grigsby P.W. (2014). Curative radiation therapy for locally advanced cervical cancer: Brachytherapy is NOT optional. Int. J. Radiat. Oncol. Biol. Phys..

[70] Barraclough L.H., Swindell R., Livsey J.E., Hunter R.D., Davidson S.E. (2008). External beam boost for cancer of the cervix uteri when intracavitary therapy cannot be performed. Int. J. Radiat. Oncol. Biol. Phys..

[71] Dimopoulos J.C., Kirisits C., Petric P., Georg P., Lang S., Berger D., Potter R. (2006). The Vienna applicator for combined intracavitary and interstitial brachytherapy of cervical cancer: Clinical feasibility and preliminary results. Int. J. Radiat. Oncol. Biol. Phys..

[72] Liu Z.S., Guo J., Zhao Y.Z., Lin X., Zhang B.Y., Zhang C., Wang H.Y., Yu L., Ren X.J., Wang T.J. (2017). Computed Tomography-Guided interstitial Brachytherapy for Locally Advanced Cervical Cancer: Introduction of the Technique and a Comparison of Dosimetry with Conventional Intracavitary Brachytherapy. Int. J. Gynecol. Cancer.

[73] Dang Y.Z., Li P., Li J.P., Bai F., Zhang Y., Mu Y.F., Li W.W., Wei L.C., Shi M. (2018). The Efficacy and Late Toxicities of Computed Tomography-based Brachytherapy with intracavitary and interstitial Technique in Advanced Cervical Cancer. J. Cancer.

[74] Wang W., Zhang F., Hu K., Hou X. (2018). Image-guided, intensity-modulated radiation therapy in definitive radiotherapy for 1433 patients with cervical cancer. Gynecol. Oncol..

[75] Mohamed S., Kallehauge J., Fokdal L., Lindegaard J.C., Tanderup K. (2015). Parametrial boosting in locally advanced cervical cancer: Combined intracavitary/interstitial brachytherapy vs. intracavitary brachytherapy plus external beam radiotherapy. Brachytherapy.

[76] Frohlich G., Vizkeleti J., Nguyen A.N., Major T., Polgar C. (2019). Comparative analysis of image-guided adaptive interstitial brachytherapy and intensity-modulated arc therapy versus conventional treatment techniques in cervical cancer using biological dose summation. J. Contemp. Brachytherapy.

[77] Narayan K., van Dyk S., Bernshaw D., Khaw P., Mileshkin L., Kondalsamy-Chennakesavan S. (2014). Ultrasound guided conformal brachytherapy of cervix cancer: Survival, patterns of failure, and late complications. J. Gynecol. Oncol..

[78] Sinnatamby M., Nagarajan V., Kanipakam Sathyanarayana R., Karunanidhi G., Singhavajala V. (2016). Study of the dosimetric differences between (192)Ir and (60)Co sources of high dose rate brachytherapy for breast interstitial implant. Rep. Pract. Oncol. Radiother..

[79] Kumar M., Thangaraj R., Alva R.C., Koushik K., Ponni A., Janaki M.G. (2020). Interstitial high-dose-rate brachytherapy using cobalt-60 source for cervical cancer: Dosimetric and clinical outcomes from a single institute. J. Contemp. Brachytherapy.

[80] Tiwari R., Narayanan G.S., Narayanan S., Suresh Kumar P. (2020). Long-term effectiveness and safety of image-based, transperineal combined intracavitary and interstitial brachytherapy in treatment of locally advanced cervical cancer. Brachytherapy.

[81] Fallon J., Park S.J., Yang L., Veruttipong D., Zhang M., Van T., Wang P.C., Fekete A.M., Cambeiro M., Kamrava M. (2016). Long term results from a prospective database on high dose rate (HDR) interstitial brachytherapy for primary cervical carcinoma. Gynecol. Oncol..

[82] Murakami N., Kobayashi K., Shima S., Tsuchida K., Kashihara T., Tselis N., Umezawa R., Takahashi K., Inaba K., Ito Y. (2019). A hybrid technique of intracavitary and interstitial brachytherapy for locally advanced cervical cancer: Initial outcomes of a single-institute experience. BMC Cancer.

[83] Le Guyader M., Kee D.L.C., Thamphya B., Schiappa R., Gautier M., Chand-Fouche M.E., Hannoun-Levi J.M. (2022). High-dose-rate brachytherapy boost for locally advanced cervical cancer: Oncological outcome and toxicity analysis of 4 fractionation schemes. Clin. Transl. Radiat. Oncol..

